# Increased *BDNF* methylation in saliva, but not blood, of patients with borderline personality disorder

**DOI:** 10.1186/s13148-018-0544-6

**Published:** 2018-08-22

**Authors:** Mara Thomas, Nora Knoblich, Annalena Wallisch, Katarzyna Glowacz, Julia Becker-Sadzio, Friederike Gundel, Christof Brückmann, Vanessa Nieratschker

**Affiliations:** 1 Department of Psychiatry and Psychotherapy, University Hospital Tübingen, Calwerstr. 14, 72076 Tübingen, Germany; 2 Graduate Training Centre of Neuroscience, University of Tübingen, Tübingen, Germany

**Keywords:** Epigenetics, Saliva, DNA methylation, BPD, DBT, Biomarker, Treatment outcome, BDNF

## Abstract

**Background:**

The importance of epigenetic alterations in psychiatric disorders is increasingly acknowledged and the use of DNA methylation patterns as markers of disease is a topic of ongoing investigation. Recent studies suggest that patients suffering from Borderline Personality Disorder (BPD) display differential DNA methylation of various genes relevant for neuropsychiatric conditions. For example, several studies report differential methylation in the promoter region of the brain-derived neurotrophic factor gene (*BDNF*) in blood. However, little is known about *BDNF* methylation in other tissues.

**Results:**

In the present study, we analyzed DNA methylation of the *BDNF* IV promoter in saliva and blood of 41 BPD patients and 41 matched healthy controls and found significant hypermethylation in the BPD patient’s saliva, but not blood. Further, we report that *BDNF* methylation in saliva of BPD patients significantly decreased after a 12-week psychotherapeutic intervention.

**Conclusions:**

Providing a direct comparison of *BDNF* methylation in blood and saliva of the same individuals, our results demonstrate the importance of choice of tissue for the study of DNA methylation. In addition, they indicate a better suitability of saliva for the study of differential *BDNF* methylation in BPD patients. Further, our data appear to indicate a reversal of disease-specific alterations in *BDNF* methylation in response to psychotherapy, though further experiments are necessary to validate these results and determine the specificity of the effect.

**Electronic supplementary material:**

The online version of this article (10.1186/s13148-018-0544-6) contains supplementary material, which is available to authorized users.

## Background

Borderline personality disorder (BPD) is a severe mental disorder that is characterized by instability in affect, interpersonal relationships, and self-image, in addition to impulsivity, fear of abandonment, anger, and self-mutilating behavior [1]. The estimated lifetime prevalence of BPD is 1.6–5.9%, as estimated by two large nonclinical surveys in the USA [2, 3]. However, despite its high prevalence, the pathogenesis and underlying biological mechanisms of BPD are not fully understood. According to the biosocial developmental model of BPD proposed by M. Linehan in 1993, the susceptibility for the disorder is enhanced by an early emotional vulnerability, which is then potentiated across the life span. Initial vulnerability is mainly caused by environmental risk factors such as childhood abuse or neglect. The estimated contribution of genetic factors to the disorder is in the range of 42–68% [4, 5], while environmental factors account for the remaining variance.

Recent evidence indicates that the interplay of environmental and genetic factors in the development of psychiatric disorders is partially mediated by epigenetic regulation [6, 7]. Epigenetic modifications induce changes in gene expression without altering the DNA sequence. One of the most prominent and best studied epigenetic mechanisms is DNA methylation, a covalent modification of cytosine in a cytosine-guanine-dimer (CpG site). Although DNA methylation is generally described as a silencing epigenetic mark, it is increasingly acknowledged that its effect on gene expression is context-dependent. Hence, it may induce silencing of a gene, when found within its promoter region, but enhance expression, when found in the gene body [8, 9]. The degree of DNA methylation at a specific locus is determined by the underlying DNA sequence [10] and is to some extent dynamically regulated by DNA methyltransferase enzymes. As these act in response to environmental stimuli [11], DNA methylation provides the cell with a way to adapt to changes in the environment [12] and is an ideal candidate mechanism for studying the interplay of genetic and environmental signals on disease development [13]. A major challenge in epigenetic research is the cell type and tissue specificity of DNA methylation [14]. As access to brain tissue is limited, the great majority of epigenetic studies in psychiatry are conducted with blood as surrogate tissue [15].

In line with this, DNA methylation signatures have been analyzed in the peripheral blood of several BPD patient cohorts. Using targeted approaches aimed at well-known psychiatric candidate genes, epigenetic dysregulation in the blood of BPD patients has been reported e.g., for the serotonin receptor 2A (*HTR2A*), the monoamine oxidase A and B (*MAOA* and *MAOB*), the soluble catechol-o-methyltransferase (*S-COMT*), the glucocorticoid receptor (*GR/NR3C1*) (all reported by [16]), and the brain-derived neurotrophic factor (*BDNF*) [17]. Further, hypothesis-free epigenome-wide studies revealed a number of novel candidate genes to be differentially methylated in patients suffering from BPD [18, 19]. However, the findings for most of the above-mentioned studies are not fully consistent with each other, and their significance yet remains to be determined by replication in independent cohorts. For the role of *BDNF* methylation in BPD, support is already available from a study conducted by Thaler et al. [20], showing that increased *BDNF* methylation in patients with bulimic eating behavior is particularly prominent when associated with comorbid BPD. In addition, Thaler et al. and Perroud et al. [17] had found an association of *BDNF* methylation with childhood trauma. In line with these findings, several independent studies report a link between *BDNF* methylation, stress, and trauma [21–23]. Here, the most convincing evidence is available for animal models of early-life stress (ELS) [24–26]. For example, Roth et al. [26] report increased methylation of the *BDNF* gene in the prefrontal cortex of rats exposed to abusive mothers. In humans, post-traumatic stress disorder in combat veterans [22] and exposure to domestic violence in women [21] have been associated with increased BDNF methylation in peripheral blood. Further, a recent study found that DNA methylation within the *BDNF* gene moderates the association between childhood trauma and depressive symptoms [23]. It is hypothesized that the link between BDNF and psychological stress is mediated via the crosstalk of neurotrophin and glucocorticoid pathways [27], as BDNF signaling is a target of the glucocorticoid stress response [28]. Hence, the high prevalence of ELS among patients with BPD [29] makes it difficult to disentangle its effects on DNA methylation from BPD-specific effects. Another confounder for epidemiologic studies of *BDNF* methylation is smoking. Next to its reported global effects on DNA methylation [30, 31], there is evidence for the association of prenatal smoke exposure with changes in offspring *BDNF* methylation and expression [32]. These alterations may be long-lasting and promote vulnerability to psychiatric disease later in life, as suggested by human [33] and animal studies [34, 35]. With regard to direct effects of smoking on *BDNF* expression, most studies indicate increased peripheral BDNF protein in smokers as compared to non-smokers [36–38], but there are no reports of altered *BDNF* methylation.

Adding even further to the difficulty of studying BPD-specific effects, *BDNF* methylation was also found associated with a broad range of psychiatric symptoms and disorders other than BPD, such as bipolar disorder [39, 40], depression [41], schizophrenia [42], and suicidality [43, 44] (reviewed in [45, 46]). The ubiquitous role of *BDNF* methylation in psychiatry is presumably caused by the broad expression of the BDNF protein in the brain, its importance in learning and memory [47] and its key regulating function in neuronal differentiation, and neurite and synaptic growth [48]. *BDNF* promoter hypermethylation, as reported in the vast majority of studies, should lead to a decreased expression of the protein. Indeed, this is what independent studies of patient cohorts report for depression [49, 50], bipolar disorder [39, 51], schizophrenia [52] and, most interestingly, also for BPD [53] (for general review see [54]). In addition, an increasing number of studies suggest that antidepressant and mood-stabilizing substances increase *BDNF* expression in the blood [55–57] and brain [58, 59]. In line with this, D’Addario et al. [39] reported that mood-stabilizing medication decreases *BDNF* exon I promoter methylation. These findings provide further indication that low *BDNF* expression plays a role in the pathophysiology of BPD.

In 2013, Perroud et al. reported that psychotherapeutic treatment alone (dialectical behavior therapy) leads to a reversion of the initially increased DNA methylation of *BDNF* promoter I and IV in BPD patients [17]. The effect was specific for those patients that had responded with significant alleviation of symptoms to the therapy [17]. This indicates that *BDNF* methylation may serve as biomarker for symptom severity in BPD patients and as an indicator of treatment success. Epigenetic biomarker have already been proposed as both predictors and correlates of symptom improvement in PTSD patients [60] and would also be highly desirable for BPD patients, where they could pave the way towards personalized treatment. In addition to *BDNF* methylation, DNA methylation of *APBA3* (amyloid beta A4 precursor protein-binding family A member 3) and *MCF2* (oncogene MCF2) has recently been proposed as blood-based biomarker for BPD patients. In this case, methylation at the respective genes was proposed as predictor of therapy response [61].

However, recent evidence indicates that saliva might be a superior surrogate tissue to blood for the study of DNA methylation in psychiatric disorders. Cross-tissue comparisons show that saliva mirrors methylation levels in the brain to a greater extent than blood does [62]. This was explicitly shown for a number of CpG sites within *BDNF* [63], even though the explanatory power of the respective study is limited as data on the brain tissue did not originate from the same study cohort from which blood and saliva was sampled. In addition, salivary biomarkers display a much more convenient, non-invasive, and safe method for studying DNA methylation alterations. As such, saliva-based epigenetic biomarkers are universally applicable in in- and out-patient settings.

So far, differential *BDNF* methylation initially found in blood [39, 41], was confirmed to be also present in saliva for bipolar disorder [64], anxiety and depression [65, 66], but has not been investigated for BPD yet. For that reason, we assessed *BDNF* promoter IV methylation in both saliva and blood from the same BPD patients, thereby enabling a direct comparison of methylation levels in both tissues. Further, since Perroud et al. [17] had reported that dialectical behavior therapy (DBT), one of the most frequently applied psychotherapeutic intervention for BPD patients [67], leads to a decrease of previously elevated *BDNF* methylation levels in BPD patients, we sought to replicate this finding by reassessing the blood and salivary *BDNF* methylation in a subsample of patients after a 12-week DBT.

## Results

### Study population

BPD patients and healthy controls did not differ significantly in age, sex, and alcohol consumption. However, there were significantly more habitual smokers in the group of BPD patients (see Table 1 for details). Further, 85.36% of all BPD patients were under current psychopharmacological medication at the time of sampling, as opposed to 0% in the healthy control group. BPD patients scored significantly higher in the BSL23 (Borderline Symptom List 23), SCL90R (Symptom Checklist-90-revised), and Childhood Trauma Questionnaire (CTQ).

**Table 1:** Comparison of BPD patient and healthy control cohorts

	BPD patients (T1)	Healthy controls	*p* value
*N*	41	41	
Age	30.4 ± 8.6	30.7 ± 9.3	n.s.
Proportion of women (%)	85.36%	85.36%	n.s.
Proportion of individuals who are			
Habitual smokers	53.65%	9.76%	< 0.001
Habitual drinkers	85.37%	95.12%	n.s.
Under current medication*	85.36%	0%	< 0.001
Psychiatric questionnaires			
GSI score (SCL90R)	2.1 ± 0.54	0.27 ± 0.24	< 0.001
PST score (SCL90R)	69.88 ± 9.15	17.8 ± 13	< 0.001
BSL23 total score	2.48 ± 0.76	0.17 ± 0.23	< 0.001
CTQ total score	62.9 ± 24.4	33.93 ± 8.47	< 0.001

Age and results from self-administered psychiatric questionnaires are displayed as mean ± standard deviation. *p* values derive from statistical analysis with independent *t* test or chi-square test for comparison of percentages

*Psychopharmacological medication only

*n.s.* not significant (*p* value > 0.05)

### Higher *BDNF* IV promoter methylation levels in saliva, but not blood, of BPD patients as compared to healthy controls

**Fig. 1:**
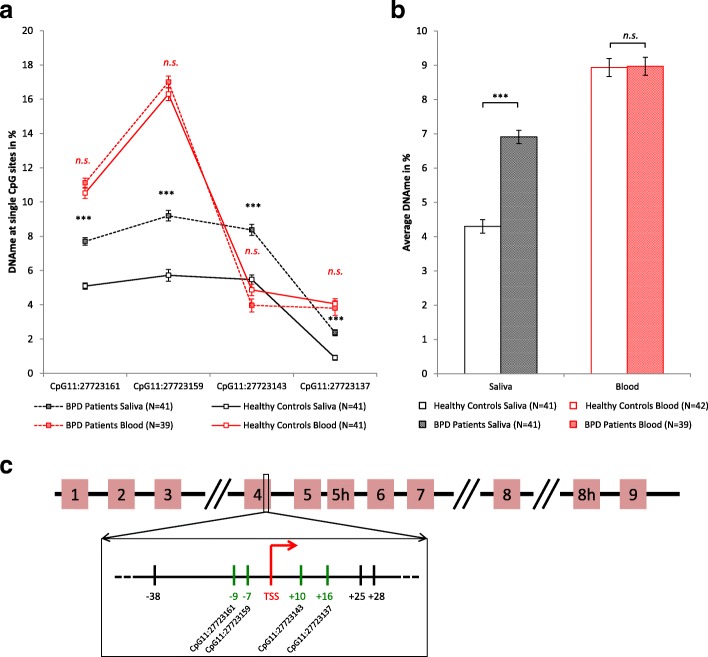
**a**, **b** DNA methylation at the *BDNF* IV promoter in blood and saliva of BPD patients (T1) (*N* = 41 for saliva, *N* = 39 for blood) as compared to healthy controls (*N* = 41). Data shown for single CpG sites (**a**) and average methylation calculated over all analyzed CpG sites (**b**), error bars represent SEM. Three asterisks (***) indicate statistical significance at *p* value < 0.001. **c** Schematic drawing of the *BDNF* gene with exons 1–9. Analyzed CpG sites (marked in green) are within the exon IV promoter, in direct vicinity of the transcription start site (TSS)

In saliva samples, DNA methylation was significantly higher in BPD patients than in healthy controls at all four analyzed CpG sites within the *BDNF* IV promoter (*p* < 0.001 for all sites, Fig. 1, see Table 2 for details). Further, the average methylation level calculated from all analyzed sites was higher in BPD patients than in healthy controls (M = 6.9%, SE = 0.19 vs. M = 4.3%, SE = 0.20, M = mean, SE = standard error). This difference, − 2.6%, 95% CI [− 3.163, − 2.061] was significant (*t* (80) = − 9.431, *p* value = 1.26 × 10^−14^) and represented a large effect (Cohen’s *d* = 2.1). These differences between BPD patients and healthy controls were also significant after including smoking behavior and experience of ELS as covariates into a general linear model to predict DNA methylation (*b* = 2.33, SE = 0.38, 95% CI [1.571, 3.058], *β* = 0.65, *t* (78) = 6.123, *p* value = 3.46 × 10^−8^ for average methylation). Neither covariate had a significant influence on DNA methylation in the model for any of the analyzed CpG sites (see Additional file 1: Table S2, for detailed results) and their addition to the model resulted in an average change in estimate (CIE) of 3.4% (smoking) and 10.2% (ELS). In DNA isolated from whole blood, *BDNF* methylation levels did not differ significantly between BPD patients and healthy controls neither for single CpG sites, nor for the average calculated from all sites (patient average 9.0% vs. healthy controls average 8.9%, detailed data in Additional file 1: Table S1). In line with this, multiple regression analysis showed no effect of group, smoking, or ELS on the blood DNA methylation at all analyzed CpG sites (see Additional file 1: Table S2 for detailed results).

**Table 2:** Statistics for the comparison of saliva *BDNF* methylation in BPD patients and healthy controls

CpG site	Mean difference	CI lower	CI upper	*p* value	Cohen’s *d*
CpG11:27723161	− 2.6	− 3.181	− 2.047	3.966 × 10^−14^	2.1
CpG11:27723159	− 3.5	− 4.392	− 2.556	6.637 × 10^−10^	1.7
CpG11:27723143	− 2.9	− 3.725	− 2.064	9.387 × 10^−9^	0.9
CpG11:27723137	− 1.5	− 1.922	− 1.007	1.110 × 10^−8^	1.5
Average	− 2.6	− 3.163	− 2.061	1.255 × 10^−14^	2.1

Results of independent *t* test for salivary *BDNF* IV promoter methylation in BPD patients (T1) and healthy controls. Results shown for individual CpG sites and average calculated from all sites

### No correlation between the blood and salivary *BDNF* IV methylation

**Fig. 2:**
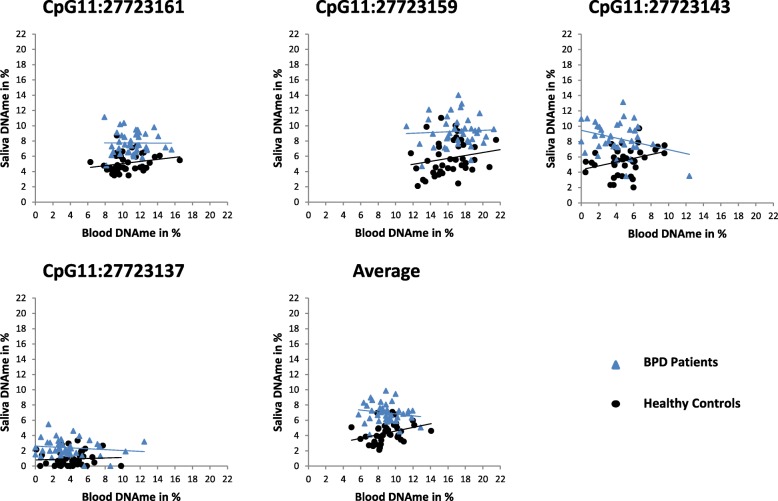
Correlation of methylation levels between blood and saliva DNA samples of BPD patients (*N* = 39) and healthy controls (*N* = 41). Regression lines are displayed separately for healthy controls and BPD patients

We compared *BDNF* IV promoter methylation in the saliva and blood of patients and controls and did neither find any significant correlation (Pearson’s correlation coefficient) in the combined cohort (*N* = 80) nor in the BPD patient cohort alone (*N* = 39). In the healthy cohort (*N* = 41), CpG11:27723143 and the average methylation were significantly but weakly correlated between both tissues (CpG11:27723143: *r* = 0.33, *p* = 0.035, 95% CI [0.088, 0.515]; average methylation: *r* = 0.33, *p* = 0.036, 95% CI [0.096, 0.574]) (Fig. 2, see Additional file 1: Table S4 for all data).

### Decrease of salivary DNA methylation levels in BPD patients following psychotherapeutic intervention

Following psychotherapeutic intervention, patients (*N* = 26) showed a significant reduction in general and BPD-specific psychiatric symptoms, as assessed by SCL90R and BSL23, respectively (Table 3).

**Table 3:** Psychiatric symptoms of BPD patients before and after DBT

	Before treatment	After treatment	*p* value
GSI score (SCL90R)	2.03 ± 0.46	1.49 ± 0.67	< 0.001
PST score (SCL90R)	69.0 ± 8.79	59.31 ± 14.29	0.001
BSL23 total score	2.29 ± 0.75	1.86 ± 0.77	0.012

Results from self-administered psychiatric questionnaires of 26 BPD patients before and after 12-week psychotherapeutic treatment (DBT) as means ± standard deviation. *p* values derive from statistical analysis with *t* test for paired samples

**Fig. 3:**
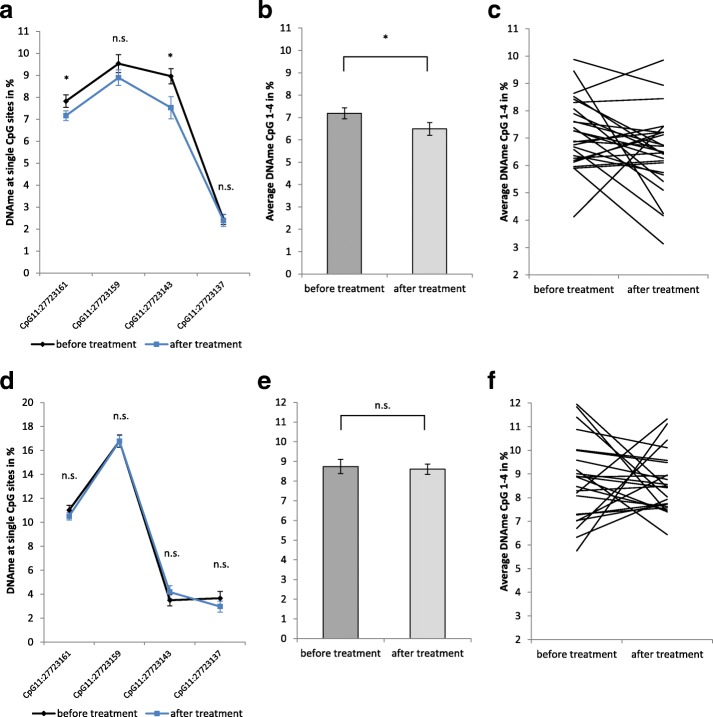
*BDNF* IV promoter methylation in BPD patients before and after a 12-week psychotherapeutic treatment in saliva (**a**–**c**, *N* = 26) and blood (**d**–**f**, *N* = 23). Data shown for single CpG sites (**a**, **d**) and for the average calculated from all analyzed CpG sites (**b**, **c**, **e**, **f**), Data points are displayed as mean ± SEM of all patients at the two time points. Statistical analysis has been conducted with paired *t* tests. Asterisks indicate significance *p* < 0.05 (**a**, **b**, **d**, **e**). Individual differences in DNA methylation levels before and after treatment shown for every patient in saliva (**c**) and blood (**f**)

After treatment, salivary DNA methylation at *BDNF* IV promoter decreased at all analyzed CpG sites (Fig. 3a–c, Table 4), though the effect was significant only for CpG11:27723161, CpG11:27723143, and the average calculated from all sites, where *BDNF* methylation decreased from 7.2 to 6.5% (mean difference = − 0.7%, SE = 0.33, 95% CI [− 1.370,-0.019], *t* (25) = − 2.118), *p* value = 0.044, Cohen’s *d* = 0.4). Analysis of changes in individual patients revealed that DNA methylation levels remained unchanged (difference less than 0.5%) in seven out of 26 patients. Of the remaining 19 patients, 14 showed decreased, and five increased methylation levels. However, the observed change in DNA methylation did not correlate with symptom reduction in individual patients (Additional file 1: Table S5). Blood DNA methylation did not change from T1 to T2, neither for single CpG sites nor for the average calculated from all sites (8.7% vs. 8.6%, mean difference = − 0.1%, SE = 0.48, 95% CI [− 1.124,0.860], *t* (22) = − 0.276, *p* value = 0.785) (Fig. 3d–f, Additional file 1: Table S3).

**Table 4:** Statistics for the comparison of saliva *BDNF* methylation in BPD patients before and after therapy

CpG site	Mean difference	CI lower	CI upper	*p* value	Cohen’s *d*
CpG11:27723161	− 0.7	− 1.303	− 0.011	0.047	0.4
CpG11:27723159	− 0.6	− 1.562	0.282	0.165	n.a.
CpG11:27723143	− 1.4	− 2.542	− 0.324	0.013	0.5
CpG11:27723137	− 0.0	− 0.660	0.561	0.871	n.a.
Average	− 0.7	− 1.370	− 0.019	0.044	0.4

Results of paired *t* tests for salivary *BDNF* IV promoter methylation in BPD patients before (T1) and after treatment (T2) (*N* = 26). Results shown for individual CpG sites and for the average calculated from all sites

*n.a.* not applicable (*p* value > 0.05)

## Discussion

We assessed *BDNF* IV promoter methylation in blood and saliva of the same individuals and found no correlation between the tissues. This has been reported previously [63], even though there is evidence for a correlation between blood and saliva methylation on a genome-wide level [62].

When comparing *BDNF* methylation in BPD patients and healthy controls, we unexpectedly did not find differences in DNA extracted from blood, as had previously been reported [17] (Table 5). One reason for this discrepancy might be that Perroud et al. [17] had analyzed the average methylation level calculated from a longer section of the *BDNF* IV promoter and did not report individual CpG methylation. Therefore, our assay might not have covered the relevant CpG sites. Further, the different methods used for DNA methylation analysis (high resolution melt analysis in [17] vs. pyrosequencing in our study) may have contributed to the conflicting results. With regard to the absolute levels of *BDNF*, the low levels of DNA methylation observed in our study (3-17%) were still higher than previously described for the respective CpG sites (< 5%) [20, 42]. Similarly, this discrepancy may be partially due to methodological differences (EpiTYPER in [20]) and ethnical differences of the study cohort (Japanese cohort in [42] as compared to Caucasian cohort in the present study). Our finding of the unaltered blood DNA methylation of the *BDNF* IV promoter in BPD patients is supported by epigenome-wide studies, which also failed to provide evidence for differential *BDNF* promoter IV methylation in the blood of BPD patients [18, 19].

**Table 5:** Genomic position of analyzed CpGs within *BDNF* IV promoter

ID	Genomic position (hg19)	Differential DNAme in the context of psychiatric disorders previously analyzed in …
CpG11:27723161	chr 11: 27,723,161–27,723,162	Blood [17, 20]and saliva [68]
CpG11:27723159	chr 11: 27,723,159–27,723,160	Blood [17, 20]and saliva [68]
CpG11:27723143	chr 11: 27,723,143–27,723,144	Blood [17, 42], saliva [68], and brain [44]
CpG11:27723137	chr 11: 27,723,137–27,723,138	Blood [17, 42] and brain [44]

In contrast to the results in blood, we found significant hypermethylation of the *BDNF* IV promoter in saliva of BPD patients as compared to healthy controls. While our study is the first to analyze *BDNF* methylation in saliva samples of BPD patients, few other studies have analyzed salivary DNA methylation at the same sites within the *BDNF* IV promoter in the context of other psychiatric conditions. These studies support an association of saliva *BDNF* hypermethylation with symptoms of psychiatric diseases [21, 65, 68]. Further, the low absolute levels of saliva *BDNF* DNA methylation observed in our study (2–10%) were comparable to those previously reported in the literature, though the exact percentage of reported methylation varies between studies (1–30%). While Moser et al. report DNA methylation levels ranging from 1 to 30% in the exon IV promoter region [21], Chagnon et al. observed levels around 2–3% [65] and Januar et al. report levels between 4 and 17% [68]. Since analysis methods and study cohorts are at least partially comparable, the most likely explanation for this discrepancy is the variability in the number of CpG sites analyzed and the method of summarization of these data into reported methylation scores. However, the observed range of the saliva DNA methylation at the analyzed CpG sites in the present study is similar to the levels reported by Keller et al. [44] in human post-mortem brain tissue at the same CpG sites (5–11%). This finding further supports the significance of saliva as surrogate tissue for the brain in the study of psychiatric disorders.

Since differences in methylation between BPD patients and healthy controls were only evident in saliva, but not blood, our findings underline the importance of considering tissue-specificity of DNA methylation in biomarker studies. Salivary DNA derives from exfoliated epithelial cells and leukocytes, which migrate from the blood stream to the oral cavity [69]. Both cell types are known to express *BDNF*, though within leukocytes, all *BDNF* expression is driven by lymphocytes [70]. As there is indication for an enrichment of lymphocytes in oral samples as compared to blood [69], differential epigenetic regulation of this particular cell type may be more evident in saliva than in blood. In addition, the observed effects may also be driven by epithelial cells, as submandibular serous and ductal cells are sources of salivary BDNF protein [71] and may therefore be dynamically regulated. Further, *BDNF* overexpression derived from salivary glands was found to influence BDNF levels in the blood and hippocampus and exert anxiolytic effects on behavior [72]. This supports a functional role for *BDNF* methylation in salivary epithelial cells. Additional studies are necessary in order to determine whether the observed alterations in salivary *BDNF* IV promoter methylation are accompanied by changes in *BDNF* expression and how these relate to psychological symptoms of BPD. However, the past years of research have shown that even small alterations in DNA methylation (Δ < 5%), as have been observed in this study, can be functionally relevant, i.e., exert influence on gene transcription [73, 74]. In fact, previous studies report similarly small changes in *BDNF* methylation associated with psychiatric symptoms (Δ = 0.42% in [65], Δ = 5.4% in [68]), and these findings are in accordance with the understanding of the multifactorial origin of psychiatric disorders [75]. In addition, the subtle differences in methylation in *BDNF* DNA methylation may also reflect the “tip of the iceberg,” i.e., the measurable output of a complex, masked pattern of stronger, cell type-specific differential methylation. In this case, effects may be driven by buccal epithelial cells or different leukocyte subtypes contained in the saliva, as previously described in more detail. However, the detailed mechanisms underlying the differential *BDNF* IV promoter methylation in BPD patients are irrelevant to the validity of the epigenetic signal as biomarker for the disorder. With regard to that, it is important to note that despite the small absolute change, the difference in methylation between BPD patients and controls is significant and the effect size was large (Cohen’s *d* = 2.1).

BDNF mRNA and protein levels were not assessed in the present study, and are difficult to assess reliably in the saliva, where protein levels are below the detection limit [76]. However, there is evidence for reduced levels of serum BDNF protein in BPD patients from previous studies [53], which is what would be expected as consequence of *BDNF* promoter hypermethylation [77]. In particular, the CpG sites analyzed in the present study are in close vicinity (− 49, − 51, − 65 and − 74 bp) to the binding site of transcription factor cAMP response element binding protein (CREB, half consensus sequence “CGTCA” [78]). CREB controls *BDNF* transcription in a DNA methylation-dependent manner [77], indicating a plausible effect of the observed methylation difference on gene expression. Further support for the relevance of *BDNF* IV promoter hypermethylation for BPD is provided by animal experiments. These show that disruption of *BDNF* IV promoter-dependent expression results in deficits in prefrontal signaling [79–81], neurobiological changes which are also observed in BPD [82].

Lastly, we found that the level of salivary, but not blood *BDNF* IV methylation significantly decreases after patients underwent a 12-week psychotherapeutic treatment. This is particularly interesting, since the hereof predicted biological consequence, increased expression of *BDNF*, is also observed in response to antidepressant and mood-stabilizing pharmacological treatment and clinical improvement of BPD [55–59]. BPD patients did not experience any change of pharmacological treatment immediately before and during study participation. Therefore, the observed effects are unlikely to derive from psychopharmacological treatment and may present a true effect of psychotherapy. Our results are consistent with the data obtained by Perroud et al. [17], showing a decrease in *BDNF* IV methylation in BPD patients after the same psychotherapeutic intervention, though they observed the effect in the blood and not saliva. However, while Perroud et al. found the effect to be specific for treatment responders, we did not find differences in methylation change between patients with and without significant improvement of psychological symptoms after therapy. Still, the finding indicates psychotherapy-induced changes in DNA methylation. Therefore, it provides support for the conceptual premise that psychotherapeutic intervention alters biological mechanisms in a way that is comparable to pharmacological treatment [83]. Nevertheless, our results need to be interpreted with caution and the specificity of the observed effect remains to be elucidated. A major limitation of both the above-mentioned previous and the current study is the lack of appropriate control groups at the second time point of sampling, i.e., BPD patients and healthy controls without psychotherapeutic intervention. Further, potential bias may have been introduced by the cellular composition of our samples. This should be addressed in future experiments by analysis of isolated cell types, inclusion of cell counts, or application of post hoc statistical deconvolution algorithms if epigenome-wide methylation data is available [84]. One of the major confounders of cellular composition of the saliva is age [85] . However, as our sample of BPD patients and healthy control individuals was matched for age, we can exclude this as a confounder and assume that differences in cellular composition in the saliva introduced random noise rather than a systematic bias to the data. Variables that indeed differed significantly between patient and control group and have a known influence on *BDNF* IV methylation are smoking, experience of ELS, and intake of pharmacological medication. Smoking is known to exert a broad influence on genome-wide DNA methylation with so far limited understanding of gene-specific effects [30, 31]. We found only little influence of current smoking behavior on *BDNF* DNA methylation. However, we did not assess prenatal exposure to smoking, though it is reported to have an effect on *BDNF* methylation, as well as promote vulnerability to BPD later in life [86]. Consequently, we cannot exclude the influence of prenatal exposure to smoking on the observed differences in the saliva *BDNF* methylation of BPD patients. In addition, there is evidence that ELS, such as childhood maltreatment and abuse, influences *BDNF* IV promoter methylation specifically [24–26] and may therefore have introduced systematic bias in the results. In line with this, the experience of ELS was identified as confounder in our linear model to predict DNA methylation, even though its influence was relatively small (10.2% CIE, *β* < 0.1). Therefore, we are not able to fully disentangle the effects of ELS and BPD on *BDNF* IV promoter methylation. Further studies will be necessary to elucidate the potential role of *BDNF* methylation in mediating vulnerability to borderline personality traits conferred by ELS [29]. With regard to intake of medication, the majority of the literature points towards a positive effect of psychopharmacological treatment on BDNF levels both in serum of patients [55, 56] as well as in cell culture experiments [58, 59, 87]. According to the DNA methylation paradigm, this increase of BDNF protein levels would correspond to a decrease in promoter methylation. Since we observed increased methylation in pharmacologically treated subjects, we therefore assume that it is more likely that medication intake has masked potential differences, rather than produced them. Further, medication did not change between T1 and T2 and is therefore unlikely to have caused the observed decrease in DNA methylation in BPD patients in response to treatment. A limitation of our study is the undefined positive predictive power of our findings, since only a limited amount of data on effect sizes for differential *BDNF* methylation in BPD was available a priori. Therefore, even though the size of our sample is in the range of, if not higher than the sample sizes reported from comparable studies (see [16, 17, 19]), the biological relevance of our finding needs to be determined in future studies and the robustness of our findings need to be confirmed by replication in independent cohorts. Further, the so far limited understanding of the dynamics of DNA methylation patterns in human peripheral tissues is increasingly investigated [88] and future studies remain to determine the stability of the observed methylation differences over time and its potential correlation or predictive value for the long-term development of psychiatric symptoms.

## Conclusions

We assessed DNA methylation levels at four sites within the *BDNF* IV promoter in blood and, for the first time, saliva of BPD patients and healthy controls and found significant hypermethylation in saliva, but not blood. Further, we found that the level of salivary, but not blood *BDNF* IV methylation significantly decreases after patients underwent a 12-week psychotherapeutic treatment. As such, our study adds to a growing body of evidence for an epigenetic dysregulation of *BDNF* in BPD, even though the previously reported differential methylation in blood [17] was not evident in our study population. Further, our results highlight the importance of considering tissue-specific differences in DNA methylation and suggest the exploration of saliva-based epigenetic biomarkers in psychiatry. Our study is the first to support the validity of *BDNF* IV promoter hypermethylation as a biomarker for BPD in a tissue other than the blood and provides additional indication for the reversal of disease-associated DNA methylation patterns in response to psychotherapy.

## Methods

### Study population

Forty-one currently hospitalized BPD patients and 41 healthy controls without any history of psychiatric disorders were included in the study. All subjects were of Caucasian origin and both groups were matched for age and sex. BPD patients were diagnosed according to the International Personality Disorder Examination (IPDE) and met at least five diagnostic criteria of BPD as defined in the fourth edition of the Diagnostic and Statistical Manual of Mental Disorders (DSM-IV). All study participants were phenotypically characterized with the following self-report questionnaires: Symptom Checklist 90 (SCL90R) [89], Borderline Symptom List 23 (BSL23) [90], and Childhood Trauma Questionnaire (CTQ) [91]. Further questionnaires assessed demographic information along with information about nicotine and alcohol consumption (AUDIT and Fagerström-Test). GSI (global severity index = average rating given to all items) and PST scores (positive symptom total = number of symptoms/items rated higher than zero) were calculated from SCL90R. Twenty-six BPD patients completed a 12-week psychotherapeutic treatment program (dialectical behavior therapy, DBT) and for these patients, psychological symptoms were assessed a second time after completion of the program using SCL90R and BSL23. Parts of the study cohort are identical to the cohort used in [61] but only those patients with available saliva samples were included and additional patients and controls were included in the present study.

### Sampling and DNA extraction

Within the first week of hospital admission (T1), saliva was collected from 41 BPD patients using the Oragene Discover DNA Collection Kit (DNA Genotek, Ottawa, Canada). Saliva from 41 control individuals was collected immediately after study inclusion using the same method. All saliva samples were stored at − 20 °C until further analysis. Venous blood was drawn from 39 of the BPD patients at T1 and from all 41 controls, collected in ethylenediaminetetraacetic acid (EDTA) tubes and stored at − 80 °C until further analysis. From the 26 patients that completed the 12-week psychotherapeutic treatment (DBT), a second saliva sample was collected during the last week of the program (T2). A second blood sample (T2) was available from 23 of these 26 patients. DNA extraction was performed using the prepIT DNA extraction Kit (DNA Genotek) for the saliva and QIAamp DNA Blood Maxi-Kit (Qiagen, Hilden, Germany) for the blood samples.

### DNA methylation analysis

Five hundred nanograms genomic DNA was bisulfite converted using the EpiTect Fast Bisulfite Conversion Kit (Qiagen) and the region of interest within the *BDNF* IV promoter was amplified using the PyroMark PCR Kit (Qiagen) according to the manufacturer’s instructions. PCR and sequencing primer (Metabion, Planegg, Germany) were as follows: PCR forward primer, 5′- TTT GTT GGG GTT GGA AGT GAA AAT-3′ PCR reverse primer, Biotin-5′-CCC ATC AAC TAA AAA CTC CAT TTA ATC TC-3′ (as in [92]); and sequencing primer, 5′-GTG GAT TTT TAT TTA TTT TTT TAT TTA T-3′.

Successful amplification as well as specificity of the PCR products was verified via agarose gel electrophoresis. Several PCR runs were performed as technical replicates for each sample (minimum two replications). Processing of the PCR amplicons for pyrosequencing analysis was performed according to the manufacturer’s protocol and PCR products were then sequenced using the PyroMark Q24 system and the PyroMark GoldReagents (Qiagen). The level of methylation in every sample was quantified using the PyroMark Q24 software version 2.0.6 (Qiagen). The pyrosequencing assay contained six CpG sites within the *BDNF* IV promoter, but only four sites passed pyrosequencing quality control and were used for further analysis (see Table 1 and Fig. 1c for genomic position). Only samples with standard deviation of < 3% between technical replicates were included in the analysis. DNA methylation standards with 0%, 25%, 50%, 75%, and 100% methylation (Qiagen) were used to generate a standard curve and all measurements were calibrated accordingly (see Additional file 1: Figure S1). In all steps of the DNA methylation analysis (bisulfite conversion, PCR, and pyrosequencing), samples were processed in balanced design in order to avoid batch effects.

### Statistical analysis

Statistical analysis was performed with SPSS (IBM, version 26). Group mean comparisons were performed with Student’s *t* test. In addition, multiple regression analysis was performed to test the effect of group (BPD T1 vs. healthy controls) on DNA methylation, including smoking behavior (smoker vs. non-smoker) and ELS (CTQ total score) as covariates. Before and after treatment comparisons were performed using paired two-sided Student’s *t* test. Cohen’s effect size *d* for *t* test comparisons was calculated from the z-score. Differences in percentages between groups were assessed with chi-square test. Bivariate correlation analysis was performed using Pearson’s correlation coefficient and 95% percentile bootstrapping was performed.

## Additional file

Additional file 1: **Table S1.** Results of independent *t* test for blood *BDNF* IV promoter methylation in BPD patients (T1) and healthy controls. Results shown for individual CpG sites and average calculated from all sites. **Table S2.** Results of multiple regression analysis using group (BPD vs. healthy controls), smoking (smoker vs. non-smoker), and early-life stress (CTQ total score) as predictors and DNA methylation as dependent variable. Analysis was performed for each individual CpG site and for the average DNA methylation calculated from all analyzed CpG sites, as well as for saliva (SAL) and blood (BL). Table indicates regression coefficients b (b), standard error of b (SE b), lower and upper bound of 95% bootstrapped confidence intervals (CI lower b, CI upper b), standardized regression coefficient (*β*), *p* value (*p* value) and *R*^2^ of the model (*R*-squared). **Table S3.** Results of paired *t* test for the blood *BDNF* IV promoter methylation in BPD patients before (T1) and after treatment (T2). Results shown for individual CpG sites and average calculated from all sites. **Table S4.** Results of bivariate correlation analysis of the blood and salivary *BDNF* methylation at CpGs 1–4 and the average calculated from all sites. Ninety-five percentile bootstrapping was performed, and significant correlation (*α*=0.05) is marked in bold. **Table S5.** Correlation analysis of symptom reduction and change in salivary DNA methylation in BPD patients (*N*=26). Difference in scores of psychiatric questionnaires were correlated with difference in DNA methylation at all analyzed CpG sites using Pearson’s correlation coefficient. Table shows results of two-tailed significance test. **Figure S1.** Methylated DNA standards (0%, 25%, 50% 75%, 100%) plotted against measured methylation of BDNF-IV Pyrosequencing Assay. Regression line, formula, and coefficient of determination were produced with Excel 2010 and are shown in the graph. Raw methylation values were transformed using the linear equation. Resulting negative values were set to zero. (DOCX 93 kb)

## Abbreviations

BDNF: Brain-derived neurotrophic factor
BPD: Borderline personality disorder
BSL23: Borderline Symptom List 23
cAMP: Cyclic adenosine monophosphate
CIE: Change in estimate
CpG: Cytosine-phosphate-guanine
CREB: cAMP response element binding protein
CTQ: Childhood Trauma Questionnaire
DBT: Dialectical behavior therapy
DNA: Desoxyribonucleic acid
DNAme: DNA methylation
DSM-IV: Diagnostic and Statistical Manual of Mental Disorders IV
EDTA: Ethylenediaminetetraacetic acid
ELS: Early-life stress
GR/NR3C1: Glucocorticoid receptor
GSI: Global Severity Index
HTR2A: Serotonin receptor 2A
IPDE: International Personality Disorder Examination
MAOA: Monoamine oxidase A
MAOB: Monoamine oxidase B
PCR: Polymerase chain reaction
PST: Positive Symptom Total
SCL90R: Symptom Checklist-90-revised
S-COMT: Soluble catechol-O-methyltransferase
