# Supplementary material for: Increased BDNF methylation in saliva, but not blood, of patients with borderline personality disorder
Source: Clin Epigenetics. 2018 Aug 22;10:109. doi: 10.1186/s13148-018-0544-6 (PMC6106893; doi:10.1186/s13148-018-0544-6)
Supplement: Supplementary file 1 — Table S1. Results of independent t test for blood BDNF IV promoter methylation in BPD patients (T1) and healthy controls. Results shown for individual CpG sites and average calculated from all sites. Table S2. Results of multiple regression analysis using group (BPD vs. healthy controls), smoking (smoker vs. non-smoker), and early-life stress (CTQ total score) as predictors and DNA methylation as dependent variable. Analysis was performed for each individual CpG site and for the average DNA methylation calculated from all analyzed CpG sites, as well as for saliva (SAL) and blood (BL). Table indicates regression coefficients b (b), standard error of b (SE b), lower and upper bound of 95% bootstrapped confidence intervals (CI lower b, CI upper b), standardized regression coefficient (β), p value (p value) and R2 of the model (R-squared). Table S3. Results of paired t test for the blood BDNF IV promoter methylation in BPD patients before (T1) and after treatment (T2). Results shown for individual CpG sites and average calculated from all sites. Table S4. Results of bivariate correlation analysis of the blood and salivary BDNF methylation at CpGs 1–4 and the average calculated from all sites. Ninety-five percentile bootstrapping was performed, and significant correlation (α=0.05) is marked in bold. Table S5. Correlation analysis of symptom reduction and change in salivary DNA methylation in BPD patients (N=26). Difference in scores of psychiatric questionnaires were correlated with difference in DNA methylation at all analyzed CpG sites using Pearson’s correlation coefficient. Table shows results of two-tailed significance test. Figure S1. Methylated DNA standards (0%, 25%, 50% 75%, 100%) plotted against measured methylation of BDNF-IV Pyrosequencing Assay. Regression line, formula, and coefficient of determination were produced with Excel 2010 and are shown in the graph. Raw methylation values were transformed using the linear equation. Resulting negative values were s [file 13148_2018_544_MOESM1_ESM.docx]

**Additional file 1**

Table S1: Results of independent T-Test for blood *BDNF* IV promoter methylation in BPD patients (T1) and healthy controls. Results shown for individual CpG sites and average calculated from all sites.

| **CpG Site** | **Mean difference** | **CI lower** | **CI upper** | **p-value** | **Cohen’s d** |
| --- | --- | --- | --- | --- | --- |
| CpG11:27723161 | -0.6 | -1.423 | 0.218 | 0.148 | n.a. |
| CpG11:27723159 | -0.7 | -1.756 | 0.344 | 0.185 | n.a. |
| CpG11:27723143 | -0.9 | -0.123 | 1.933 | 0.084 | n.a. |
| CpG11:27723137 | 0.3 | -0.797 | 1.305 | 0.632 | n.a. |
| Average | -0.0 | -0.786 | 0.711 | 0.921 | n.a. |

Table S2: Results of multiple regression analysis using group (BPD vs. healthy controls), smoking (smoker vs. non-smoker) and early life stress (CTQ total score) as predictors and DNA methylation as dependent variable. Analysis was performed for each individual CpG site and for the average DNA methylation calculated from all analyzed CpG sites, as well as for saliva (SAL) and blood (BL). Table indicates regression coefficients b (b), standard error of b (SE b), lower and upper bound of 95% bootstrapped confidence intervals (CI lower b, CI upper b), standardized regression coefficient (β), p-value (p-value) and R^2^ of the model (R squared).

|  |  |  | b | SE b | CI lower bound | CI upper bound | β | p-value | Tolerance | VIF | R^2^ |
| --- | --- | --- | --- | --- | --- | --- | --- | --- | --- | --- | --- |
| SAL | CpG11:277723161 | Constant | 5,021 | 0,339 | 4,346 | 5,695 |  | **2,232E-24** |  |  | 0,515 |
|  |  | GROUP | 2,615 | 0,393 | 1,832 | 3,397 | 0,716 | **3,646E-09** | 0,536 | 1,866 |  |
|  |  | NIC | -0,171 | 0,352 | -0,872 | 0,530 | -0,044 | 6,283E-01 | 0,772 | 1,295 |  |
|  |  | ELS | 0,003 | 0,008 | -0,013 | 0,019 | 0,033 | 7,493E-01 | 0,604 | 1,656 |  |
|  | CpG11:277723159 | Constant | 5,422 | 0,548 | 4,331 | 6,512 |  | **1,986E-15** |  |  | 0,419 |
|  |  | GROUP | 3,141 | 0,636 | 1,876 | 4,407 | 0,582 | **4,323E-06** | 0,536 | 1,866 |  |
|  |  | NIC | 0,213 | 0,569 | -0,920 | 1,346 | 0,037 | 7,090E-01 | 0,772 | 1,295 |  |
|  |  | ELS | 0,008 | 0,013 | -0,018 | 0,034 | 0,071 | 5,257E-01 | 0,604 | 1,656 |  |
|  | CpG11:277723143 | Constant | 5,195 | 0,494 | 4,212 | 6,178 |  | **1,250E-16** |  |  | 0,386 |
|  |  | GROUP | 2,487 | 0,573 | 1,347 | 3,628 | 0,526 | **4,181E-05** | 0,536 | 1,866 |  |
|  |  | NIC | 0,472 | 0,513 | -0,549 | 1,493 | 0,093 | 3,600E-01 | 0,772 | 1,295 |  |
|  |  | ELS | 0,007 | 0,012 | -0,016 | 0,030 | 0,068 | 5,554E-01 | 0,604 | 1,656 |  |
|  | CpG11:277723137 | Constant | 0,507 | 0,268 | -0,026 | 1,040 |  | 6,220E-02 |  |  | 0,367 |
|  |  | GROUP | 1,069 | 0,311 | 0,450 | 1,687 | 0,423 | **9,355E-04** | 0,536 | 1,866 |  |
|  |  | NIC | 0,148 | 0,278 | -0,406 | 0,702 | 0,055 | 5,963E-01 | 0,772 | 1,295 |  |
|  |  | ELS | 0,011 | 0,006 | -0,001 | 0,024 | 0,209 | 7,479E-02 | 0,604 | 1,656 |  |
|  | AVRG | Constant | 4,036 | 0,328 | 3,384 | 4,688 |  | **5,574E-20** |  |  | 0,534 |
|  |  | GROUP | 2,328 | 0,380 | 1,571 | 3,085 | 0,647 | **3,464E-08** | 0,536 | 1,866 |  |
|  |  | NIC | 0,166 | 0,340 | -0,512 | 0,843 | 0,043 | 6,281E-01 | 0,772 | 1,295 |  |
|  |  | ELS | 0,007 | 0,008 | -0,008 | 0,023 | 0,094 | 3,494E-01 | 0,604 | 1,656 |  |
| BL | CpG11:277723161 | Constant | 10,476 | 0,499 |  | 9,482 | 11,469 | **2,264E-33** |  |  | 0,027 |
|  |  | GROUP | 0,603 | 0,577 | 0,163 | -0,548 | 1,753 | 3,001E-01 | 0,523 | 1,913 |  |
|  |  | NIC | -0,095 | 0,516 | -0,024 | -1,124 | 0,934 | 8,547E-01 | 0,744 | 1,344 |  |
|  |  | ELS | 0,002 | 0,012 | 0,019 | -0,022 | 0,025 | 8,978E-01 | 0,593 | 1,687 |  |
|  | CpG11:277723159 | Constant | 16,500 | 0,632 |  | 15,241 | 17,760 | **1,114E-39** |  |  | 0,042 |
|  |  | GROUP | 1,172 | 0,732 | 0,249 | -0,285 | 2,630 | 1,134E-01 | 0,523 | 1,913 |  |
|  |  | NIC | -0,769 | 0,655 | -0,153 | -2,073 | 0,535 | 2,438E-01 | 0,744 | 1,344 |  |
|  |  | ELS | -0,004 | 0,015 | -0,036 | -0,034 | 0,027 | 8,055E-01 | 0,593 | 1,687 |  |
|  | CpG11:277723143 | Constant | 4,503 | 0,618 |  | 3,272 | 5,733 | **2,532E-10** |  |  | 0,059 |
|  |  | GROUP | -1,487 | 0,715 | -0,320 | -2,912 | -0,062 | 4,105E-02 | 0,523 | 1,913 |  |
|  |  | NIC | 0,710 | 0,640 | 0,143 | -0,564 | 1,984 | 2,707E-01 | 0,744 | 1,344 |  |
|  |  | ELS | 0,009 | 0,015 | 0,085 | -0,021 | 0,038 | 5,571E-01 | 0,593 | 1,687 |  |
|  | CpG11:277723137 | Constant | 4,284 | 0,637 |  | 3,016 | 5,552 | **2,826E-09** |  |  | 0,011 |
|  |  | GROUP | 0,098 | 0,737 | 0,021 | -1,370 | 1,566 | 8,944E-01 | 0,523 | 1,913 |  |
|  |  | NIC | -0,422 | 0,659 | -0,085 | -1,735 | 0,891 | 5,240E-01 | 0,744 | 1,344 |  |
|  |  | ELS | -0,005 | 0,015 | -0,053 | -0,036 | 0,025 | 7,237E-01 | 0,593 | 1,687 |  |
|  | AVRG | Constant | 8,941 | 0,455 |  | 8,035 | 9,846 | **1,545E-31** |  |  | 0,001 |
|  |  | GROUP | 0,097 | 0,526 | 0,029 | -0,952 | 1,145 | 8,548E-01 | 0,523 | 1,913 |  |
|  |  | NIC | -0,144 | 0,471 | -0,041 | -1,082 | 0,793 | 7,598E-01 | 0,744 | 1,344 |  |
|  |  | ELS | 0,000 | 0,011 | 0,004 | -0,021 | 0,022 | 9,790E-01 | 0,593 | 1,687 |  |

Table S3: Results of paired T-Test for blood *BDNF* IV promoter methylation in BPD patients before (T1) and after treatment (T2). Results shown for individual CpG sites and average calculated from all sites.

| **CpG Site** | **Mean difference** | **CI lower** | **CI upper** | **p-value** | **Cohen’s d** |
| --- | --- | --- | --- | --- | --- |
| CpG11:277231611 | -0.5 | -1.638 | 0.619 | 0.360 | n.a. |
| CpG11:27723159 | -0.0 | -1.373 | 1.355 | 0.990 | n.a. |
| CpG11:27723143 | 0.7 | -0.847 | 2.221 | 0.363 | n.a. |
| CpG11:27723137 | -0.7 | -2.22 | 0.829 | 0.354 | n.a. |
| Average | -0.132 | -1.124 | 0.860 | 0.785 | n.a. |

Table S4: Results of bivariate correlation analysis of blood and salivary *BDNF* methylation at CpGs 1-4 and the average calculated from all sites. 95% percentile bootstrapping was performed, significant correlation (α=0.05) is marked in bold.

| **Group** | **N** | **Statistic** | **CpG11:**  **27723161** | **CpG11:**  **27723159** | **CpG11:**  **27723143** | **CpG11:**  **27723137** | **AVRG** |
| --- | --- | --- | --- | --- | --- | --- | --- |
| All | 80 | Pearson Correlation Coefficient | 0,184 | 0,204 | -0,140 | -0,073 | 0,069 |
|  |  | Significance (two-sided) | 0,102 | 0,070 | 0,215 | 0,522 | 0,543 |
|  |  | 95% CI lower bound | -0,019 | 0,009 | -0,331 | -0,306 | -0,111 |
|  |  | 95% CI upper bound | 0,397 | 0,403 | 0,054 | 0,177 | 0,266 |
| Healthy controls | 41 | Pearson Correlation Coefficient | 0,213 | 0,210 | **,330^*^** | 0,089 | **,328^*^** |
|  |  | Significance (two-sided) | 0,180 | 0,187 | 0,035 | 0,580 | 0,036 |
|  |  | 95% CI lower bound | -0,016 | -0,067 | 0,088 | -0,238 | 0,096 |
|  |  | 95% CI upper bound | 0,451 | 0,480 | 0,515 | 0,425 | 0,574 |
| BPD patients | 39 | Pearson Correlation Coefficient | -0,011 | 0,053 | -0,307 | -0,138 | -0,168 |
|  |  | Significance (two-sided) | 0,946 | 0,749 | 0,057 | 0,401 | 0,307 |
|  |  | 95% CI lower bound | -0,366 | -0,246 | -0,616 | -0,459 | -0,485 |
|  |  | 95% CI upper bound | 0,364 | 0,350 | 0,118 | 0,191 | 0,144 |

Table S5: Correlation analysis of symptom reduction and change in salivary DNA methylation in BPD patients (N=26). Difference in scores of psychiatric qestionnaires were correlated with difference in DNA methylation at all analyzed CpG sites using Pearson’s correlation coefficient. Table shows results of two-tailed significance test.

| **Δ Score** | **Statistic** | **Δ CpG11:**  **27723161** | **Δ CpG11:**  **27723159** | **Δ CpG11:**  **27723143** | **Δ CpG11:**  **27723137** | **Δ Average** |
| --- | --- | --- | --- | --- | --- | --- |
| GSI | Pearson's r | 0,069 | 0,123 | 0,076 | -0,268 | 0,029 |
|  | p-value | 0,738 | 0,55 | 0,712 | 0,185 | 0,889 |
| PST | Pearson's r | 0,068 | 0,027 | 0,066 | -0,261 | -0,007 |
|  | p-value | 0,741 | 0,898 | 0,747 | 0,198 | 0,975 |
| BSL23 | Pearson's r | 0,068 | 0,022 | -0,103 | -0,27 | -0,08 |
|  | p-value | 0,74 | 0,916 | 0,615 | 0,182 | 0,698 |


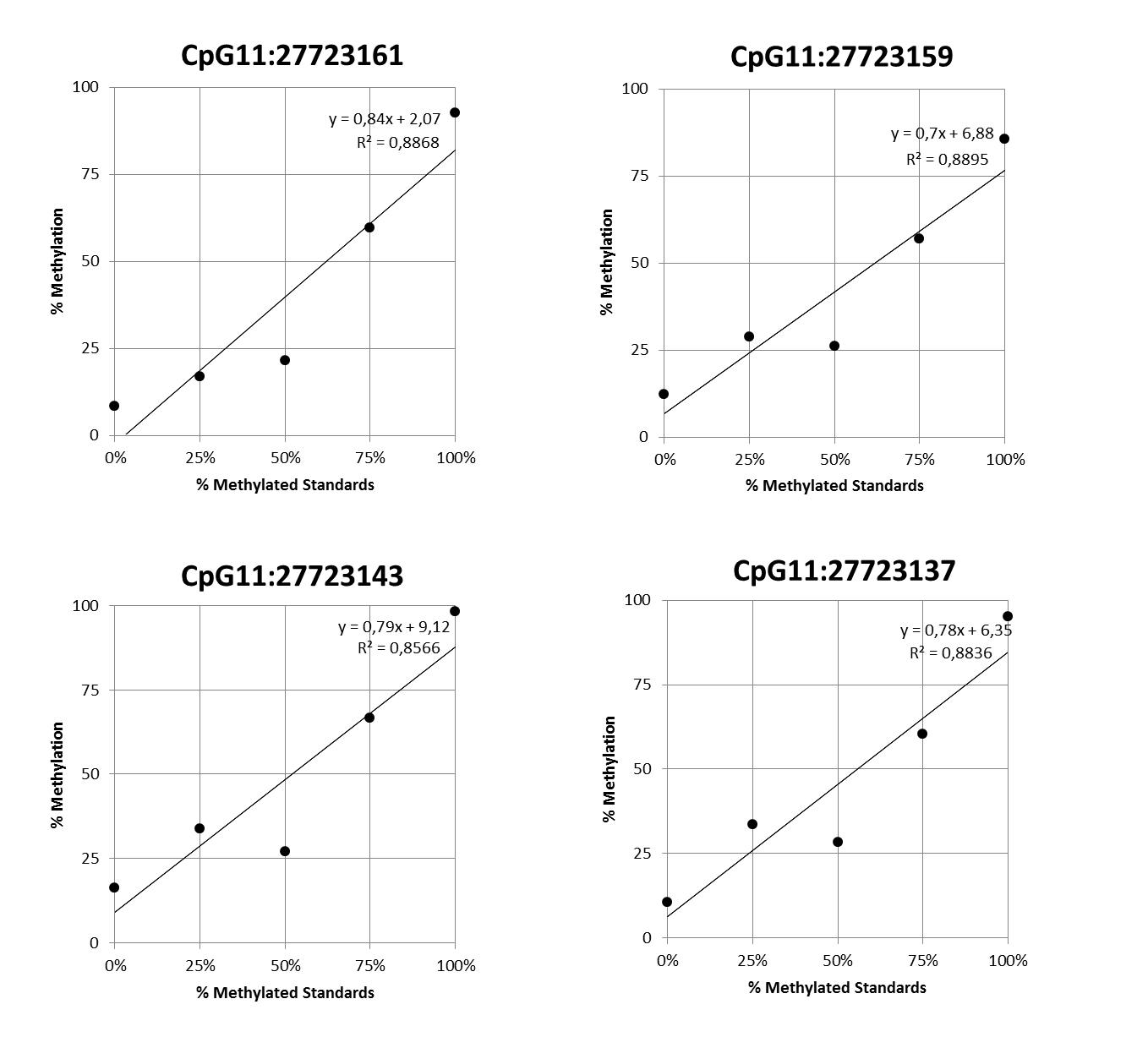


Figure S1: Methylated DNA standards (0%, 25%, 50% 75%, 100%) plotted against measured methylation of BDNF-IV Pyrosequencing Assay. Regression line, formula and coefficient of determination were produced with Excel 2010 and are shown in the graph. Raw methylation values were transformed using the linear equation. Resulting negative values were set to zero.
